# Modeling Williams syndrome from a neurodevelopmental perspective: recent advances, model-based translational insights and future directions

**DOI:** 10.1007/s12519-026-01020-x

**Published:** 2026-03-17

**Authors:** Ya-Yue Chen, Wei-Jun Chen, Rui Zhang, Chai Ji, Yu-Han Zhang, Da-Qing Ma, Qiao-Juan Shi, Yi-Cheng Xie

**Affiliations:** 1 Laboratory Animal Center, Perioperative and Systems Medicine Laboratory, Department of Child Health Care, Children’s Hospital, National Clinical Research Center for Children and Adolescents’ Health and Diseases, Zhejiang University School of Medicine, Hangzhou 310052, China; 2 Zhejiang Key Laboratory of Neonatal Diseases, Hangzhou 310052, China; 3 Zhejiang Center of Laboratory Animals, Hangzhou Medical College, Hangzhou 310013, China; 4 Division of Anesthetics, Pain Medicine and Intensive Care, Department of Surgery and Cancer, Faculty of Medicine, Imperial College London, Chelsea and Westminster Hospital, London, SW10 9NH UK

**Keywords:** Mouse models, Neurodevelopmental disorder, Organoids, Williams syndrome

## Abstract

**Background:**

Williams syndrome (WS; OMIM #194,050) is a multisystem pediatric genetic disorder caused by a heterozygous microdeletion of a 1.5–1.8 Mb region at chromosome 7q11.23, encompassing 26 to 28 genes. Clinical hallmarks include cardiovascular anomalies, distinctive craniofacial morphology and neurodevelopmental deficits characterized by hypersociability, cognitive impairment and anxiety. Although causative therapies for WS still remain elusive, advances in gene editing and forebrain organoids have already greatly furthered our understanding of the underlying mechanisms.

**Data sources:**

This narrative review was conducted by searching for papers using PubMed/MEDLINE. Relevant publications were identified using single and/or combined keywords including: Williams syndrome, 7q11.23, microdeletion, microduplication, atypical deletion, neurodevelopment, neuroanatomy, neuroimaging. cognitive impairment, mouse models, *GTF2I*, *GTF2IRD1*, *CLIP2*, *LIMK1*, *NCF1, EIF4H, STX1A/B, FZD9, HIP1, CLDN3, FKBP6,* organoid, induced pluripotent stem cell (iPSC) and forebrain organoids.

**Results:**

Mouse models including multigene deletion strains recapitulating the WS critical region and single-gene knockout strains targeting *Gtf2i*, *Gtf2ird1*, *Clip2* and *Limk1* replicate key WS neurodevelopmental phenotypes, substantially contributing to mechanistic studies and therapeutic screening. In addition, forebrain organoids derived from patients or generated by gene editing have provided human-specific insights into progenitor dynamics, synaptic function, and ribosome biogenesis.

**Conclusions:**

This review synthesizes recent progress in WS modeling in the context of neurodevelopmental impairments. While animal models and forebrain organoids have substantially accelerated both mechanistic understanding and translational research in WS, effective diagnostic and therapeutic approaches are still unavailable. Integration of animal models and forebrain organoids, together with the advanced technologies, will be essential for biomarker discovery and development of mechanism-based therapeutic approaches.

**Graphical Abstract:**

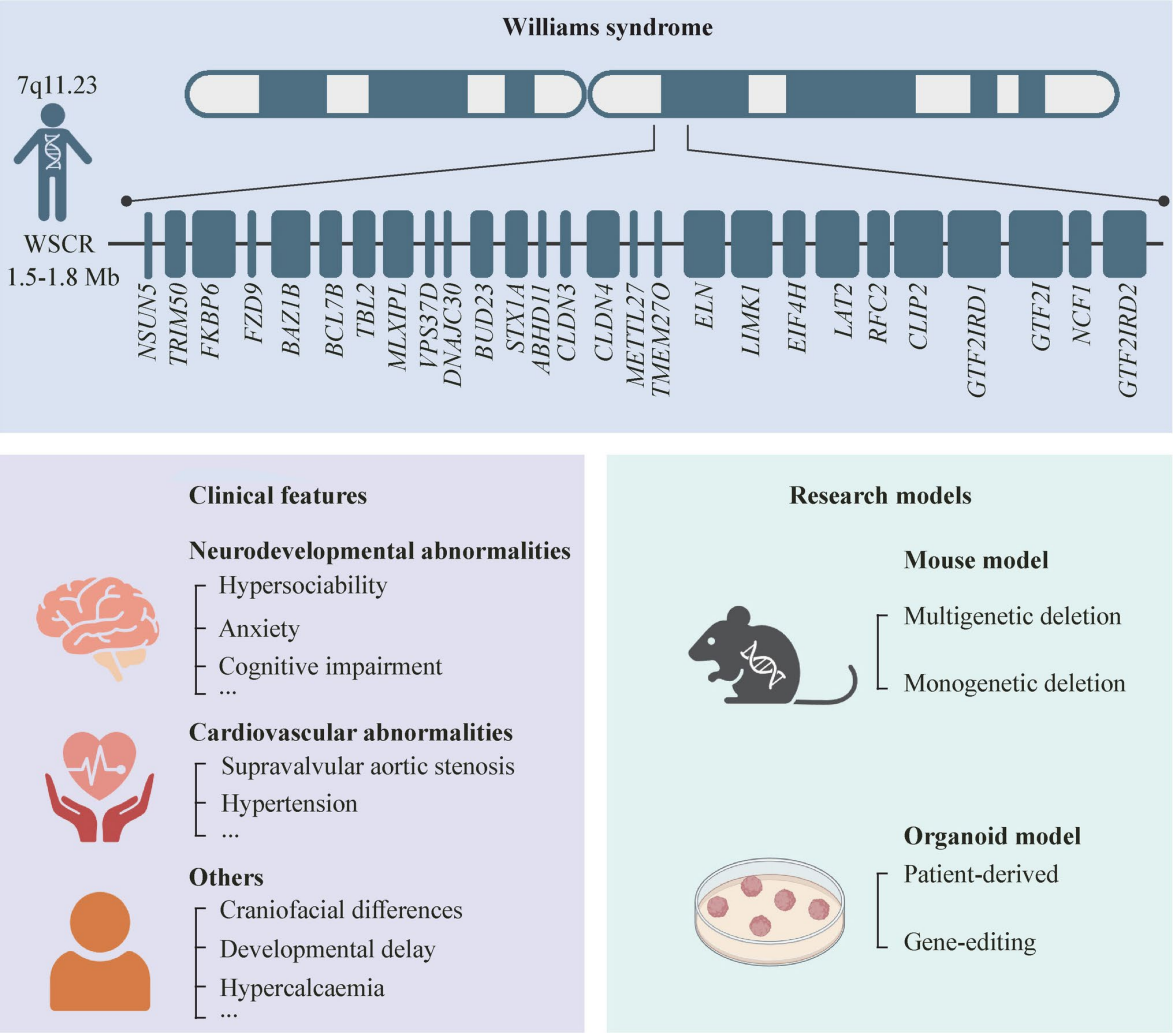

## Introduction

Williams syndrome (WS; OMIM#194,050) is a multisystem hereditary pediatric disorder caused by a heterozygous deletion of a 1.5–1.8 Mb region on chromosome 7q11.23, affecting 26 to 28 genes. The prevalence is approximately 1 in 7500 live births [1]. The common 1.5 Mb deletion accounts for about 95% of cases, whereas a larger 1.8 Mb deletion occurs in 4%, and atypical deletions constitute the remainder [2].

Typical WS patients carrying the canonical 1.5 Mb deletion exhibit a series of neurodevelopmental impairments. Hypersociability is one of the most distinctive behavioral features of WS patients and is characterized by heightened social motivation and an increased tendency to seek out social contact. However, children with WS are reported to have difficulties sustaining friendships, probably due to poor awareness of personal space boundaries, social disinhibition, difficulty with interactive play or preference for solo play, and a lack of shared interests with peers [3–6]. In addition to hypersociability, WS patients are often diagnosed with attention deficit hyperactivity disorder, anxiety and specific phobia [7–13]. Auditory hypersensitivity has also been reported in a subset of WS patients, indicating altered sensory processing in WS patients [14–17]. More recently, an abnormal body mass index has been observed in WS patients, potentially reflecting atypical eating behaviors [18]. Mild to moderate intellectual disability is also common in WS patients. The mean intelligence quotient of WS patients usually lies between 50 and 60, with a range of 40 to 100 [19, 20]. In addition to global cognitive impairment, poor visuospatial abilities are common in WS patients [21–24]. Moreover, motor deficits are also frequently observed in WS patients, with fine motor movements being particularly affected [22, 24, 25].

**Fig. 1:**
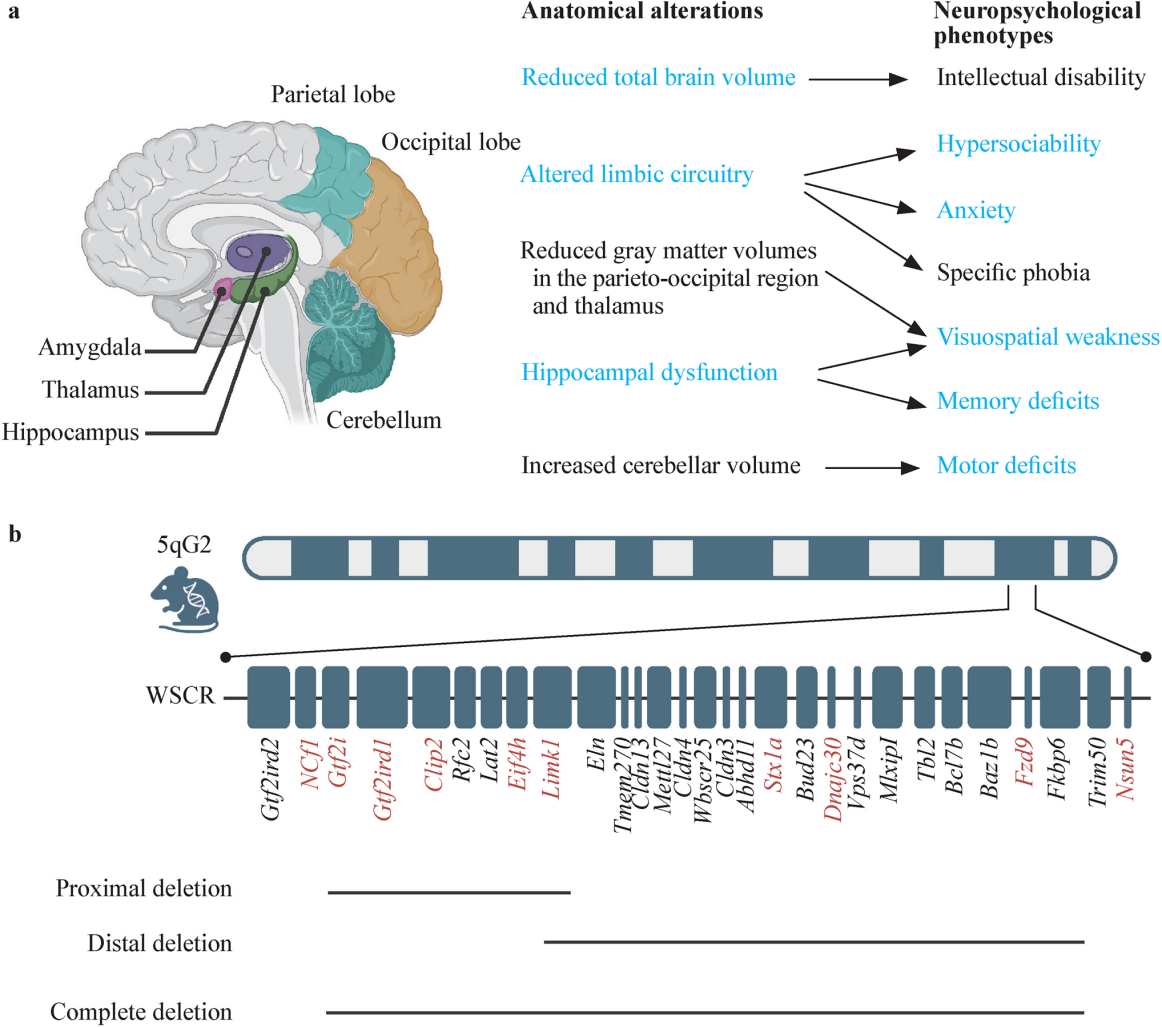
Overview of neuropsychological phenotypes in WS patients and complete deletion heterozygous mouse models of WS. **a** Summary of anatomical alterations in the brain and corresponding neuropsychological phenotypes in WS patients. Neuroanatomical regions are indicated in the left panel. The parieto-occipital region is the anatomical transition zone at the junction of the posterior parietal lobe and the anterior occipital lobe. The volumes of the parietal lobe, occipital lobe and thalamus are decreased in WS patients [27]. The arrows in the right panel indicate the correlations between anatomical alterations and neuropsychological phenotypes. The phenotypes colored in blue were confirmed in complete deletion mice. Phenotypes colored in black are only observed in WS patients; **b** WS mouse models: proximal deletion, distal deletion, and complete deletion mouse models varying gene deletions in the WSCR on chromosome 5G2, which is homologous to human 7q11.23 [64, 68] Genes colored in red are functionally linked to neurodevelopmental deficits that have been confirmed in mouse models. *WS* Williams syndrome, *WSCR* Williams syndrome critical region

Anatomical alterations, which are considered highly related to cognitive and behavioral phenotypes, have been observed in the brains of WS patients in multiple neuroimaging studies (Fig. 1a) [26]. Magnetic resonance imaging reveals a marked reduction in total brain volume of 10%–15% in WS patients, which is associated with mild to moderate intellectual disability [27–29]. After adjusting for total brain volume, individuals with WS presented reduced thalamic and parieto-occipital region gray matter volumes and reduced gray matter density in subcortical and cortical regions comprising the human visual-spatial system [23, 27, 30–32]. In addition, functional and metabolic abnormalities in the hippocampal formation have been reported to contribute to deficits in spatial navigation and long-term memory [33]. Alterations in limbic circuitry, particularly the amygdala-prefrontal circuitry, are closely linked to hypersociability and increased anxiety [27, 31, 34–40]. Interestingly, absolute cerebellar volumes were reduced in WS patients, whereas cerebellar volumes relative to intracranial volumes and the volumes of the posterior superior cerebellar vermis were significantly increased, supporting the hypothesis that volume alterations in the cerebellum are associated with motor deficits and poor short-term memory in WS patients [28, 41].

Atypical WS patients exhibit similar but distinctive cognitive and behavioral phenotypes due to genetic differences. For example, WS patients with a 1.8 Mb deletion exhibit more impaired visuospatial abilities and more severe behavioral problems than those with a 1.5 Mb deletion [42–44]. 7q11.23 duplication syndrome (7Dup; OMIM# 609,757), which is caused by the microduplication of the Williams syndrome critical region (WSCR) at chromosome 7q11.23, shares several neurodevelopmental impairments with WS, such as attention deficit hyperactivity disorder, anxiety and mild to moderate intellectual disability. However, individuals with 7q11.23 microduplication syndrome (7Dup) exhibit minor impairments in visual–motor integration compared with those with WS, and some individuals with 7Dup are affected by autism spectrum disorders [45].

Compared with other neurodevelopmental disorders, such as autism spectrum disorder, WS has distinct social and systemic features, facilitating diagnosis from other neurodevelopmental disorders [46–48]. Currently, the diagnosis of WS is primarily based on certain typical symptoms, such as valvular aortic stenosis, characteristic facial features, developmental delay, and hypercalcemia, which are ultimately confirmed by genetic testing approaches, including fluorescence in situ hybridization, polymorphic microsatellite markers, multiplex ligation-dependent probe amplification, and chromosomal microarray analysis, facilitating earlier and more accurate diagnosis of WS [49]. Early diagnosis is essential, although noninvasive prenatal testing remains unavailable. Neonatal follow-up and early neurodevelopmental assessments may facilitate timely detection [50, 51].

Cardiovascular and neurological involvement represent the most severe therapeutic challenges. Surgical interventions are currently available to address vascular anomalies, which could also benefit the treatment of neurodevelopmental disorders in WS patients [52, 53]. Some common antidepressants and anxiolytics, such as selective serotonin reuptake inhibitors and buspirone, have been clinically proven to be helpful for WS patients [54–57]. Furthermore, the methylphenidate class, particularly the osmotic-release oral system-methylphenidate class, has been shown to improve attention deficit/hyperactivity disorders in WS patients in the clinic [58, 59]. Interestingly, melatonin also appears to be beneficial for neurodevelopmental impairments in WS [60]. In addition to medication, appropriate education and training are widely used to treat WS [61]. Despite these measures, curative treatments are unavailable, and neurological impairment remains a major determinant of quality of life. Understanding the underlying mechanisms and identifying effective therapies are critical priorities.

Although clinical cohorts and patient biospecimens are essential in WS research, clinical studies face limitations due to variability, methodological constraints, and ethical issues. Thus, animal and organoid models are indispensable for mechanistic exploration and therapeutic discovery [62]. This review summarizes advances in WS modeling, emphasizes neurodevelopmental impairments, and explores translational applications.

## Multigene deletion models of Williams syndrome

To faithfully recapitulate the genetic features of WS patients, disease models with multigene heterozygous deletion models spanning the WSCR have been established. These disease models, including organoid and transgene mouse models, enable systematic investigations of disease mechanisms and provide robust platforms for preclinical drug screening and therapeutic evaluation.

### Mouse models

Genes in the WSCR are conserved between humans and mice, enabling the generation of mouse models for WS (Fig. 1b and Table 1) [63]. These models recapitulate the neurological and behavioral phenotypes of WS, supporting the findings of mechanistic studies and therapeutic screening (Table 2). Several mouse models with a microdeletion at mouse chromosome 5G2 have been developed to recapitulate the genetic features of WS patients. In 2009, Li et al. generated proximal (PD, *Gtf2i*–*Limk1*) and distal (DD, *Limk1*–*Fkbp6*) deletion strains via Cre–loxP. Both PD mice and DD mice produce double heterozygous (D/P) mice, which mimic WS deletions [64]. However, in D/P mice, *Limk1* is completely deleted rather than present at a haplo-insufficient dosage, which may lead to phenotypic differences compared with WS patients. *Limk1* encodes a serine/threonine kinase that regulates the actin cytoskeleton by phosphorylating and inactivating actin-depolymerizing factors or cofilin [65–67]. PD, DD, and D/P mice are all viable and fertile, although the D/P mice exhibit mildly reduced reproductive capacity. Similar to findings in WS patients, brain volumes are reduced by approximately 9.7% in DD mice and 14% in D/P mice, whereas PD mice show a slight reduction only in females. In both PD and D/P mice, the number of neurons in layer V of the somatosensory cortex was significantly greater than that in wild-type controls, whereas no differences were detected in other regions of the somatosensory cortex or in the visual cortex. In terms of social behavior, PD, DD, and D/P mice exhibit both shared and distinct phenotypes. For example, all three mouse models demonstrated heightened social interest in the partition test. In the social preference paradigm, PD and D/P mice spend more time in the chamber containing a conspecific. Similarly, both lines show reduced win rates in the tube test. In contrast, D/P mice performed significantly worse than both PD and DD mouse models did in the hanging wire and rotarod tests, suggesting that genes within both the proximal and distal regions contribute to motor learning and coordination (Fig. 1b and Table 2) [64].

**Table 1:** Individual deletion of genes in different mouse models

Mouse models	*Gtf2ird2*	*Ncf1*	*Gtf2i*	*Gtf2ird1*	*Clip2*	*Rfc2*	*Lat2*	*Eif4h*	*Limk1*	*Eln*	*Tmem270*	*Cldn13*
CD	+	+	−	−	−	−	−	−	−	−	−	−
PD	+	+	−	−	−	−	−	−	−	+	+	+
DD	+	+	+	+	+	+	+	+	−	−	−	−

Mouse models	*Mettl27*	*Cldn4*	*Wbscr25*	*Cldn3*	*Abhd11*	*Stx1a*	*Bud23*	*Dnajc30*	*Vps37d*	*Mlxipl*	*Tbl2*	*Bcl7b*	*Baz1b*	*Fzd9*	*Fkbp6*	*Trim50*	*Nsun5*
CD	−	−	−	−	−	−	−	−	−	−	−	−	−	−	−	+	+
PD	+	+	+	+	+	+	+	+	+	+	+	+	+	+	+	+	+
DD	−	−	−	−	−	−	−	−	−	−	−	−	−	−	−	+	+

The WSCR genes ordered by genomic position. *WSCR* Williams syndrome critical region, *CD* complete deletion, *PD* proximal deletion, *DD* distal deletion. “ + ” positive, “−” negative

**Table 2:** Phenotypes of different Williams syndrome mouse models

Variables	WS patient	CD	PD	DD	D/P	*Gtf2i*^±^	*Gft2ird1*^±^	*Gtf2ird1*^*−/−*^
Reduced brain volume	+	+	+	+	+	−	−	ND
Increased neuronal density	+	+	+	−	+	ND	ND	ND
Altered neuronal architecture	+	+	+	ND	ND	+	ND	ND
Developmental delay or learning impairment	+	+	+	+	+	−	+	+
Weakness in spatial skills	+	−	ND	ND	ND	−	−	−
Hypersociability	+	+	+	−	+	+	+	+
Hypersensitivity to sound	+	+	+	−	−	+	+	+
Anxiety	+	+	+	+	+	−	+	+
Motor deficit	+	+	+	−	+	+	+	+

*WS* Williams syndrome, *CD* Complete deletion, *PD* Proximal deletion, *DD* Distal deletion, *D/P* PD mice and DD mice produced double heterozygous, *ND* not determined. “ + ” positive, “−” negative

In 2014, a complete deletion (CD) model spanning *Gtf2i*–*Fkbp6* was generated by the Victoria Campuzano laboratory, avoiding homozygous loss of *Limk1* and thus better replicating WS [68]. Unlike D/P mice, CD mice accurately mimic the WSCR deletion in human. Like D/P mice, CD mice are viable and fertile and exhibit growth retardation and distinctive craniofacial features. Compared with that of wild-type mice, brain weight is decreased by approximately 9%, which is comparable to that of DD mice and slightly less than that of D/P mice. Notably, CD mice presented decreased cell density but preserved volume in the amygdala. In the hippocampus, mature neuron density and volume are significantly reduced in CD mice. Morphological analysis revealed reduced neuronal maturation in the brains of CD mice. Behaviorally, CD mice differ substantially from wild-type controls in both the cognitive and social domains, displaying increased startle responses to acoustic stimuli, impaired fear memory performance, and hypersociability [68].

In the following years, CD mice were widely used to study the underlying pathogenic mechanisms of WS and for preclinical drug screening [69–75]. To evaluate whether CD mice are able to model WS in children, the laboratory of Susanna Pietropaolo investigated the phenotypic features of CD mice during infancy (before 3 weeks of age) and adolescence (between 3 and 7 weeks of age). During infancy, CD mice exhibited reduced body growth, delayed sensory development, and altered patterns of ultrasonic vocalizations and exploratory behaviors. During adolescence, CD mice exhibit reduced locomotor activity, attenuated acoustic startle responses, and alterations in social interaction and communication [71]. In the conditioned fear task, CD mice also exhibit deficits in contextual fear conditioning [74]. In addition, CD mice display impairments in short-term memory rather than long-term memory, which differs from the findings in WS patients [73].

Morphological analysis further revealed decreased brain weight, reduced hippocampal and cortical spine length, and lower dendritic spine density in CA1 pyramidal neurons. These phenotypes mimic the clinical manifestations of WS in children, highlighting the potential of CD mice as an ideal model for early diagnosis and therapeutic development in WS. Moreover, several studies have targeted the neurobehavioral symptoms of WS using CD mice in the context of pharmacological screening. For example, combined daily oral administration of verapamil, an L-type calcium channel blocker, and curcumin for 5 weeks significantly improved neurodevelopmental impairments in CD mice aged between 8 and 14 weeks. Further study suggested that the combination of verapamil and curcumin altered the inflammasome-related, MAPK and PI3K/AKT signaling–related pathways and reduced the number of activated microglia. However, none of the individual treatments had any effect [75]. Daily intraperitoneal injections of JZL184 (a potent and selective monoacylglycerol lipase inhibitor) for two weeks could specifically normalize the social and cognitive phenotypes of CD mice aged between 8 and 16 weeks, suggesting that modulation of the endocannabinoid system is a promising novel therapeutic approach for WS [73]. In contrast, oxytocin failed to attenuate fear memory impairments in CD mice at 13 and 17 weeks when an oxytocin receptor antagonist was administered via intracerebroventricular infusion [74]. Taken together, these findings provide valuable mechanistic insight and preclinical evidence for developing therapies targeting neurodevelopmental impairments in WS, underscoring the translational value of CD mice as an ideal disease model of WS.

### Induced pluripotent stem cells and organoids

**Fig. 2:**
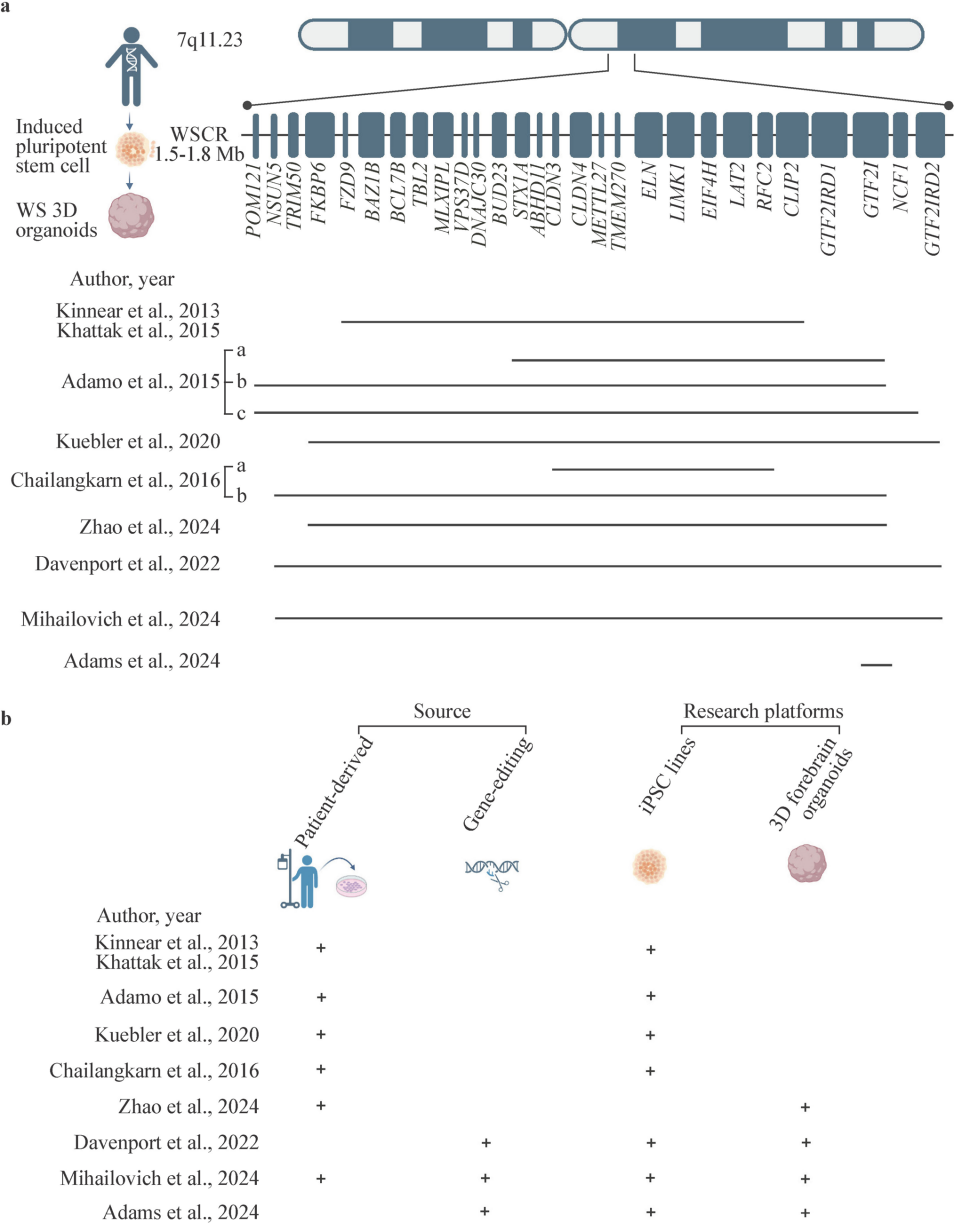
iPSC- and organoid-based models of WS. **a** Individual gene deletions in the studies reviewed. The horizontal solid lines represent the genomic regions or deletion spans investigated in each study. In the research platform of Khattak et al. (2015), the gene deletion was confirmed by qRT-PCR and the SALSA multiplex ligation probe amplification kit P029-A1 [78, 82]. iPSC line C from Adamo et al. (2015) exhibited a broader deletion beyond the *POM121*–*NCF1* region, encompassing three additional upstream genes: *SPDYE8P*, *GTF2IP1*, and *NCF18* [83]; **b** overview of research sources and platforms. The presence of a specific research source or platform is indicated by a plus sign (+) *WS* Williams syndrome, *WSCR* Williams syndrome critical region, *iPSC* induced pluripotent stem cell, *qRT-PCR* quantitative real-time polymerase chain reaction

Recent advances in induced pluripotent stem cell (iPSC) and organoid technologies have provided a novel paradigm for the study of neurodevelopmental disorders. iPSC-derived forebrain organoids preserve specific genetic mutations from WS patients and recapitulate the molecular signaling pathways involved in human neurodevelopment. In addition, iPSC-derived organoids could significantly reduce experimental timelines. Thus, iPSC-derived forebrain organoids are particularly suitable for studying the functions of species-specific human receptors and evaluating the efficacy of gene therapies [76]. Recently, iPSC-derived organoids, especially forebrain organoids, have been widely applied in mechanistic studies and drug screening in WS (Fig. 2a, b). Two major strategies have been employed in the development of WS organoid models: direct differentiation via iPSCs from WS patients and the use of gene editing to introduce WS-associated genetic deletions into iPSCs from healthy donors (Fig. 2b). Each approach offers unique advantages corresponding to specific research objectives.

**Fig. 3:**
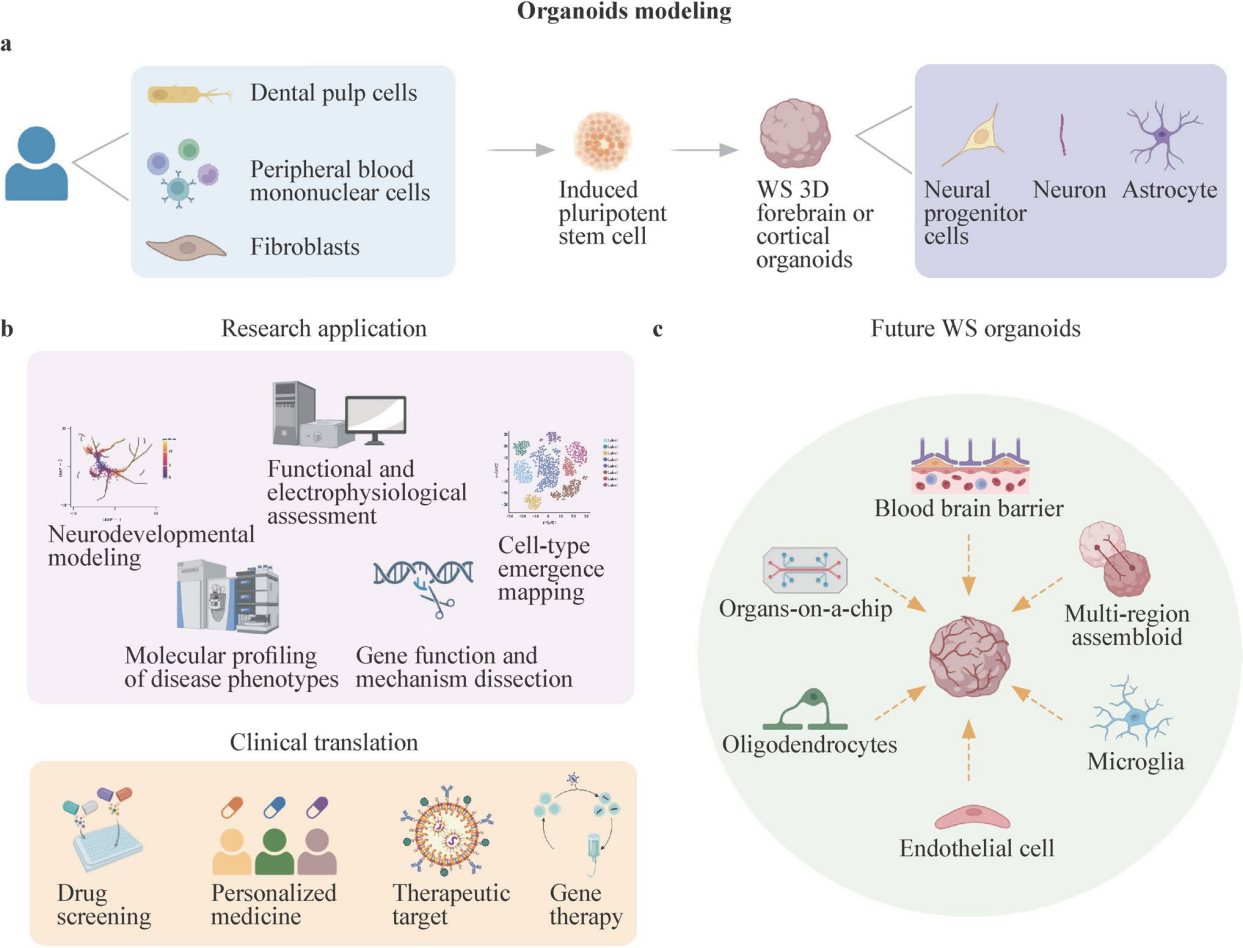
WS models constructed on the basis of brain organoids. **a** Generation of WS forebrain organoids: patient-derived somatic cells (dental pulp cells, peripheral blood mononuclear cells, fibroblasts) are reprogrammed into iPSCs and then differentiated into forebrain organoids containing neural progenitor cells, neurons, and astrocytes; **b** applications: WS organoids enable studies of neurodevelopment, cell-type specification, functional phenotyping, gene regulation, drug response evaluation, precision medicine, and gene therapy; **c** future directions: improving organoid fidelity by including microglia, oligodendrocytes, and endothelial cells, integrating organ-on-chip systems for blood–brain barrier simulation, and assembling region-specific structures for in vivo-like organization. *WS* Williams syndrome, *iPSC* induced pluripotent stem cell

Patient-derived iPSCs preserve the pathogenic mutations of WS and directly link laboratory studies with clinical research. This approach, which is widely used to model atypical WS, allows reprogramming from sources such as skin fibroblasts [77–79], dental pulp cells [80], peripheral blood mononuclear cells, and urinary cells (Fig. 3a) [81].

In 2015, Khattak et al. generated cortical neurons from WS iPSCs and reported that there was a profound alteration in action potentials, with significantly prolonged WS repolarization times and a WS deficit in voltage-activated K^+^ currents [82]. In 2016, Chailangkarn et al. reported distinct morphological alterations in layer V/VI cortical neurons differentiated from WS iPSCs, which is consistent with postmortem findings in WS patients. These findings highlight iPSC-derived cortical neurons as a reliable in vitro model for layer V/VI cortical neurons in WS [80]. More recently, Zhao et al. reported the abnormal proliferation and differentiation of neural progenitor cells in WS forebrain organoids. Transcriptome analysis revealed aberrant expression of neurodevelopmental genes in WS forebrain organoids [81].

In 2015, the Giuseppe Testa laboratory performed transcriptomic analysis on iPSC lines derived from patients with WS and 7Dup, revealing that dosage imbalances at 7q11.23 result in significant transcriptional changes in WS-associated genes. Next, Adamo et al. successfully induced the differentiation of WS iPSCs into multiple cell types, including neural progenitor cells, neural crest stem cells, and mesenchymal stem cells. Through transcriptome analysis, it was demonstrated that transcriptional dysregulation was even more severe after differentiation [83]. In 2023, the Giuseppe Testa laboratory generated patient-derived cortical organoids and reported that 7q11.23 copy number variations caused imbalances in cortical cell population composition, which is consistent with previous findings [84]. Notably, this laboratory adopted gene-editing technology in patient-derived iPSC lines, providing a novel paradigm for research in WS organoids. In 2024, this laboratory used two rounds of CRISPR/Cas9 editing to generate the wild-type control iPSC cell line isoCTL and the WS iPSC cell line isoWBS from iPSCs derived from patients with 7Dup. Next, the iPSCs were induced to differentiate into neurons, and multiomics analysis of these neurons demonstrated that ribosome biogenesis plays a key role in neurodevelopmental disorders [85]. This strategy enabled researchers to study the effects of 7q11.23 dosage variation on neurodevelopmental impairments in the same genetic background, providing a valuable research strategy for future studies.

The gene-editing approach for generating iPSCs and organoids provides a consistent genetic background, since the control iPSCs and organoids originate from the same individual, increasing the reliability of the experimental results. However, similar to animal models, a key limitation of this approach is the challenge of precisely replicating the specific mutation observed in clinical WS cases, especially atypical WS. In 2022, Davenport et al. reprogrammed dental pulp stem cells from a healthy male donor into iPSCs and used CRISPR/Cas9 to generate iPSCs with deletions spanning the *NSUN5* to *GTF2IRD2* region. These modified iPSCs were then induced into brain organoids. Transcriptome analysis revealed significant downregulation of synaptic genes and biological pathways relevant to neuronal activity, GABAergic neurons, and neurodevelopment in WS brain organoids [86].

### Single-gene deletion models of Williams syndrome

Clinical studies in individuals with typical and atypical WS have revealed probable associations between neuropsychological phenotypes and specific genes in the WSCR. However, the limited number of available studies and the variability of neuropsychological instruments make further study of the functions of candidate genes, which might be essential in neurodevelopmental impairments in WS, difficult [2]. To better understand the roles of individual genes in the WSCR, single-gene deletion models have been established, revealing the genotype‒phenotype correlation in WS.

#### *Gtf2i*

The human *GTF2I* gene encodes GTFII-I, a highly conserved and ubiquitously expressed multifunctional transcription factor. GTFII-I is involved in numerous biological processes, including embryonic development [87–89], cell cycle regulation [90–93], actin cytoskeleton dynamics, axon guidance [93], and epigenetic regulation [94, 95]. *GTF2I* is a member of the *TFII-I* gene family, which also includes *GTF2IRD1* and *GTF2IRD2*, both of which are closely associated with WS [96].

Clinical studies of individuals with atypical WS carrying smaller deletions that spare *GTF2I* and *GTF2IRD1* have provided important genotype–phenotype insights. These atypical WS patients with *GTF2I* and *GTF2IRD1* retained exhibited better visuospatial abilities and intellectual disability than typical WS patients did [97–105]. Further study revealed that intraparietal sulcus volume, which is known to be involved in visuospatial function, is decreased in typical WS patients but remains unaffected in atypical WS patients, with *GTF2I* and *GTF2IRD1* retained [100].

It has been reported that *GTF2I* dosage variations contribute approximately 10% to 20% of the observed transcriptional dysregulation in iPSC lines derived from individuals with WS and 7Dup, likely due to the disrupted interactions between *GTF2I* and the *LSD1* repressive chromatin complex [77]. In 2024, Adams et al. generated *GTF2I*-deficient iPSC lines via CRISPR/Cas9 and then induced their differentiation into neural progenitor cells, human neurons, and three-dimensional cortical organoids to investigate the role of *GTF2I* in neurodevelopment. *GTF2I*-deficient neural progenitor cells presented increased proliferation and cell cycle alterations, whereas *GTF2I*-deficient cortical organoids and neurons presented increased cell death and synaptic dysregulation. In summary, these findings suggest that *GTF2I* deficiency induces alterations in neural progenitor cells that give rise to neurons and networks characterized by impaired neuronal health and synaptic reduction [106].

The mouse gene *Gtf2i* (MGI:1,202,722), located in the proximal region of mouse chromosome 5G2, serves as the murine ortholog of human *GTF2I*. In the three different large heterozygous deletion mouse models of WS mentioned above, including PD, D/P, and CD mice, *Gtf2i* is heterozygously deleted. All three models exhibit neurobehavioral changes, including disrupted neuronal distribution, increased social interest, and impaired motor coordination, suggesting that *Gtf2i*, along with other genes located in the proximal WS region, might play critical roles in neurodevelopment [64]. The overexpression of *Gtf2i* in the hippocampus has been shown to partially rescue neurodevelopmental impairments, including hypersociability and motor deficits, in CD mice [70]. Homozygous deletion of *Gtf2i* results in embryonic lethality, whereas *Gtf2i* heterozygous mutants are viable and fertile [87, 107–109]. Although *Gtf2i* heterozygous mutants cause no significant morphological or anatomical changes in the brain, these mice exhibit prominent neurobehavioral abnormalities, including hypersociability, reduced motor coordination, increased anxiety, and auditory hypersensitivity [107, 108]. In addition, conditional deletion of *Gtf2i* in the excitatory neurons of the forebrain induces neuroanatomical defects, developmental microglial alterations, fine motor deficits, increased sociability and anxiety. Notably, pharmacological interventions such as clemastine and 4-aminopyridine, which enhance myelination, can improve these behavioral deficits [110, 111].

#### *Gtf2ird1/2*

The human *GTF2IRD1* gene encodes the protein BEN, with its murine ortholog identified as *Gtf2ird1* (MGI:1,861,942). On human chromosome 7q11.23, *GTF2I* and *GTF2IRD1* are adjacent, whereas GTF2IRD2 lies slightly downstream. As previously described, clinical studies have suggested that *GTF2IRD1* and *GTF2I* are important in maintaining normal visuospatial ability and intellectual disability. *Gtf2ird1* is extensively expressed during embryogenesis, and the maternal BEN protein can be detected in the zygote [112]. In adult mice, *Gtf2ird1* is expressed primarily in the central and peripheral nervous systems, as well as in the retina, olfactory epithelium, spiral ganglion of the cochlea, and brown adipocytes. Moderate expression is also observed in cardiac and smooth muscle tissues [113]. Both GTF2I and GTF2IRD1 have been implicated in the transcriptional regulation of known autism spectrum disorder genes [114].

To date, five distinct *Gtf2ird1* mutant mouse lines have been generated to investigate the function of GTF2IRD1 [87, 113, 115–117]. Homozygous mutants commonly present with craniofacial dysmorphology, growth delay, anxiety-like behaviors, impaired motor coordination, and altered vocalizations, whereas heterozygotes generally display milder changes (Table 2) [113, 116–120]. However, only homozygous mutants from the *Gtf2ird1*^*XE465*^ line are embryonic lethal [87]. The *Gtf2ird1*^*XE465*^ line carries an insertion in intron 22, preventing the nuclear translocation and transcriptional regulatory function of BEN by disrupting the nuclear localization signal, which might be responsible for the embryonic lethality of homozygotes. Further studies identified the GTF2IRD1-TTR-ERK axis as a key regulator of neurodevelopment [81].

To further elucidate the functions of *Gtf2i* and *Gtf2ird1*, the laboratory of Joseph D. generated a mutant mouse line with simultaneous deletion of both genes on the same chromosome [121]. Double knockout of *Gtf2i* and *Gtf2ird1* produced mice with intermediate hypersociability, auditory sensitivity, anxiety-like behaviors, and motor deficits, falling between those of single mutants and CD mice [114]. Later, they reported that the overexpression of *Gtf2ird1* in CD mice could partially rescue neurodevelopmental impairments, such as motor deficits and light-aversive behavior, while hypersociability was not rescued [122]. Taken together, these findings suggest that, in addition to *Gtf2i* and *Gtf2ird1*, additional genes within the WSCR also significantly contribute to neurodevelopmental impairments in WS.

GTF2IRD2 is located on microtubules in the cytoplasm and interacts with both GTF2IRD1 and GTF2I. Notably, overexpression of GTF2IRD2 alters the subcellular localization of overexpressed GTF2IRD1 and GTF2I in COS-7 cells [123]. Although *GTF2IRD2* is deleted in only a subset of WS patients, loss of GTF2IRD2 is associated with more severe neurodevelopmental impairments [42]. *Gtf2ird2* (MGI:2,149,780) is the murine ortholog of *GTF2IRD2*. *Gtf2ird2* overexpression antagonizes the muscle fiber type shifts driven by *Gtf2ird1* overexpression, suggesting functional interplay among *TFII-I* family members [123]. However, the role of GTF2IRD2 in neurodevelopmental disorders remains underexplored. Given the structural and functional similarities among members of the *TFII-I* gene family, as well as their apparent dose-dependent effects, understanding the individual functions of *Gtf2i*, *Gtf2ird1*, and *Gtf2ird2* and the interplay among them is critical for uncovering the molecular mechanisms underlying neurodevelopmental impairments in WS.

#### *Limk1*

LIMK1, together with LIMK2, regulates actin dynamics by phosphorylating cofilin, thereby promoting actin polymerization [124]. Interestingly, impaired visuospatial constructive cognition was observed in 8 of 10 family members with an 83.6 kb deletion containing *ELN1* and *LIMK1* [125]*.* A clinical study in several different atypical WS patients suggested that *LIMK1* hemizygosity contributes to impaired visuospatial constructive cognition [125, 126]. The mouse ortholog of *LIMK1* is *Limk1* (MGI:104,572), and studies of *Limk1* mutant mice have revealed reduced levels of phosphorylated cofilin and increased cofilin activity [127]. In these mutants, a reduction in the number of upper-layer pyramidal neurons in the cortex has been reported [128], whereas axonal growth rates [129], motor axon length, and regenerative capacity are significantly increased [130]. *Limk1* deletion also leads to morphological alterations in neurons, including dendritic spine abnormalities, as well as changes in hippocampal synaptic plasticity, particularly long-term potentiation, and behavioral abnormalities, such as altered fear responses, spatial learning, and long-term memory [127, 131]. Further investigations suggest that LIMK1 regulates long-term memory via CREB rather than cofilin, suggesting a potential therapeutic mechanism for impaired long-term memory associated with *Limk1* deficiency [131]. Furthermore, LIMK1 is involved in the regulation of the immune response and platelet activation. In a murine model of ocular inflammation, *Limk1* mutant mice presented significantly weaker inflammatory responses than wild-type controls did [132]. Similarly, in a model of acute lung injury, *Limk1*-mutant mice exhibited reduced mortality, lung edema, lung microvascular permeability, and neutrophil infiltration into the lungs [133]. Thus, these findings revealed the essential role of LIMK1 in regulating endothelial cell contractility and neutrophil trafficking. In addition, *Limk1* mutant mice have defective arterial thrombosis but do not differ from wild-type mice with respect to bleeding time, suggesting that LIMK1 plays an important role in thrombosis and represents a potential target for developing antithrombotic drugs with minimal bleeding side effects [134, 135].

#### *Clip2*

*CLIP2* (also known as CLIP-115 or Cyln2) encodes the cytoplasmic linker protein CLIP-115, which is highly expressed in the inferior olive, hippocampus, and piriform cortex in the brain [136]. *CLIP2* haploinsufficiency is not sufficient to produce but might contribute to the typical cognitive defects in WS [99, 137]. *Clip2* (MGI:1,313,136) is the murine ortholog of *CLIP2*, which is highly expressed in the CA subfields of the hippocampus and moderately expressed in other brain regions, including the amygdala, cerebral cortex, and cerebellum [138]. Clip2 has been shown to participate in the dynamic regulation of microtubule growth in neurons. In *Clip2* homozygous knockout mice, both the rate and extent of microtubule elongation are significantly increased [139]. The heterozygous deletion of *Clip2* leads to mild growth retardation, enlarged ventricles, and reduced corpus callosum volume, along with impairments in motor coordination and hippocampus-dependent memory formation [105, 138].

### Other genes

In addition to the genes mentioned above, additional genes located in the WSCR have been confirmed to be associated with neurodevelopmental disorders in mouse models. For example, *Ncf1* knockout mice exhibited mild impairments in hippocampus-dependent memory [140]. Loss of *Eif4h* in mice resulted in phenotypes similar to those of CD mice, including growth retardation, reduced brain volume, altered brain morphology, decreased neuronal number and complexity, novelty-induced hyperreactive behavior and deficits in spatial memory and fear-associated memory [141]. Double mutant STX1A/B mice presented defects in axonal guidance in the spinal cord, suggesting the important role of *Stx1a* in neurodevelopment [142]. *Dnajc30* knockout mice presented with mitochondrial dysfunction, diminished morphological features of neocortical pyramidal neurons, increased anxiety and increased interest in novel social stimuli [143]. *Fzd9* homozygous mutant mice presented large increases in apoptotic cell death in the developing dentate gyrus and impaired visuospatial abilities, whereas *Fzd9* heterozygous mutant mice were intermediate between wild-type and *Fzd9* homozygous mutant mice [144]. In addition, neural progenitor cells derived from WS iPSCs exhibited impaired proliferation and increased apoptosis due to FZD9 haploinsufficiency [80]. *Nsun5* knockout mice exhibited impaired visuospatial ability and impaired oligodendrocyte precursor cells proliferation [145]. Clinical studies have reported that *HIP1* haploinsufficiency is associated with autism spectrum disorder and epilepsy [146–149]. *Hip1* knockout mice exhibit neurological phenotypes, including failure to thrive, tremor, and gait ataxia [150]. Notably, *Cldn3*, which is highly expressed in the blood-cerebrospinal fluid barrier of the choroid plexus, has been shown to be involved in the regulation of neuroinflammation, which may also contribute to the neurodevelopmental disorder phenotype of WS patients [151, 152]. Loss of *Fkbp6* in mice results in spermatogenesis deficits in males [153]. Notably, haploinsufficiency of individual genes in the WSCR does not uniformly result in relevant phenotypes. For example, *Fkpbp6* presented no phenotype unless both alleles were knocked out, suggesting that symptomatic traits could be the result of either interactions between more than one gene or physiologic differences between mice and humans.

## Perspectives and future directions

Research on WS has evolved from descriptive observations to mechanistic and translational studies. The integration of CD mice with human forebrain organoids has clarified how hemizygosity of the WSCR influences neural progenitor cell proliferation, microglial maturation and synaptic plasticity. These biological changes underlie the characteristic cognitive and behavioral profile of WS. Studies in preclinical pharmacology have demonstrated both promising and inconclusive outcomes. Interventions such as endocannabinoid modulation, clemastine, 4-aminopyridine and combined treatment with verapamil and curcumin improved neurodevelopmental impairments in mouse models, whereas manipulation of the oxytocin pathway did not help improve fear memory deficits. These results emphasize the importance of identifying the underlying mechanisms that could guide the development of therapeutic approaches. Moving forward, translational progress will depend on integrating evidence across multiple model systems. To date, mouse models remain essential for drug screening and preclinical studies, mimicking complex symptoms and enabling circuit-level investigations of the WSCR genes with tools such as stereotaxic surgery [154–156]. Moreover, organoids capture human-specific developmental features that cannot be modeled in rodents. Together, these models provide complementary tools for mechanistic discovery, therapeutic screening, and genetic rescue experiments.

However, some limitations remain in mouse models and organoids. The WSCR is inverted between human chromosome 7q11.23 and mouse chromosome 5G2, and broader differences in brain structure and developmental trajectories limit its translation to patients. For example, cerebellar volumes are enlarged in WS patients but unaffected in D/P mice [28, 64]. In addition, developmental microglial alterations have been found in a mouse model, which might be related to neurodevelopmental impairments in WS patients [111]. However, further exploration of the underlying mechanism is difficult since there are important differences between murine microglia and their human counterparts [157]. On the other hand, organoids remain limited by a lack of vascularization and immune components, restricting their application in modeling cardiovascular phenotypes and neuroinflammation. Moreover, the lack of vascularization in human brain organoids could lead to hypoxia, thus restricting nutrient delivery and limiting tissue maturation, especially in long-term cultures [158–160]. Owing to the rarity of clinical samples and the difficulty of obtaining genetically matched controls, larger cohorts are needed to reduce variability and strengthen conclusions. In addition, the reliance on single-sex iPSC lines in some studies may also reduce generalizability.

Technological advancements make the future of organoid technology promising. By coculturing human brain organoids with blood vessel organoids, neural-specific blood-vessel networks can be formed within human brain organoids, although cell–cell communication and its outcomes require further investigation [161]. In addition, coculturing microglia or microglial progenitor cells with human brain organoids can generate microglia-containing human brain organoids [160]. Recently, a xenotransplantation approach has been developed that makes it possible to study mature human microglia in vivo, which might be useful for studying the role of microglia in WS in the future [162]. Microfluidic organ-on-a-chip systems, which mimic the blood–brain barrier and fluid shear stress, not only enhance the physiological relevance of cerebral organoids but also offer new strategies for improving drug permeability prediction (Fig. 3b). In the future, the integration of CRISPR-edited isogenic control lines with organ-on-a-chip systems, which introduce physiologically relevant parameters such as fluid shear stress, may substantially improve the fidelity and translational relevance of WS organoids (Fig. 3c) [85, 163].

In addition to mouse models and organoids, rat, domestic dog, and non-human primate models could be used in WS studies in the future. Compared with mice, rats have advanced learning and memory capacities, as well as more complex social behaviors, which are more suitable for studying neurodevelopmental impairments in WS patients than are mice. Domestic dogs are hypersociable compared with wolves, which likely results from the structural variations in *GTF2I* and *GTF2IRD1*. Thus, domestic dogs can serve as an ideal model to study the underlying mechanisms of hypersociablity in WS [164]. Non-human primates, such as cynomolgus and rhesus macaques, have been used to model psychiatric disease for several decades. The advantages of this paradigm include comparable cognitive skills, brain morphology, and social complexity in adult monkeys and humans [165]. These features make non-human primates particularly suitable for modeling the neurodevelopmental, higher-order cognitive, and complex behaviors of WS patients, and they have been applied in mechanistic studies and clinical translational research [166, 167]. For example, deficits in fine motor movements are frequently observed in WS patients, which are difficult to evaluate in mouse models and can be easily conducted in non-human primates [168]. The non-human primate model is also suitable for studying alterations in intelligence quotients and rhythmic abilities in WS, which is difficult to conduct in other models. In addition, neuroimaging technologies, including positron emission tomography and magnetic resonance imaging, could be applied in non-human primates to further study the underlying mechanism of WS [165]. However, generating large-fragment deletions in non-human primates remains challenging. At present, it may be more feasible to investigate WS by targeting several key genes individually in non-human primates.

As technological innovation has accelerated, emerging methods, including multiomics analysis [169–171] and optogenetics [172, 173], have become applicable to these platforms. Multiomics approaches allow the investigation of synaptic dysfunction in WS from multiple molecular dimensions and help to clarify the roles of epigenetic modifications, signal transduction and energy metabolism in shaping disease phenotypes. Spatial transcriptomics enables region-specific brain mapping and analysis of cell–cell interactions. Optogenetic techniques enable precise control of specific neuronal activity and provide direct evidence for probing circuit-level mechanisms in WS.

The next stage of research should focus on clinical trials specifically designed for WS, which should use biomarkers to improve patient stratification and incorporate developmentally appropriate endpoints. Recent neuroimaging studies in WS patients have revealed various morphometric brain differences, emphasizing the translational value of measures based on magnetic resonance imaging. Magnetic resonance imaging and diffusion tensor imaging have revealed altered brain connectivity in the absence of *Gtf2i* in neurons or myelinating glia in mouse models [174, 175]. In parallel, a recent study demonstrated altered neocortical dynamics in CD mice via electrophysiological recordings, providing quantifiable readouts potentially translatable to human neuroimaging biomarkers [176]. In addition to magnetic resonance imaging, advanced neuroimaging technologies such as magnetic resonance spectroscopy and positron emission tomography offer opportunities to probe the biochemical and molecular features of WS brain pathology, with the potential to identify pharmacologically targetable biomarkers. However, the clinical implementation of these techniques in WS remains limited, probably due to limited clinical resources [26]. Expanding the use of advanced neuroimaging in animal models, including rats and non-human primates, would therefore be critical for translational bridging. Moreover, in addition to *GTF2I*, other the WSCR genes, including *LIMK1* and *CLIP2*, which are involved in the regulation of synaptic plasticity, might play important roles in brain connectivity [127, 138]. Unrevealing the underlying mechanisms would help to reveal additional diagnostic and therapeutic targets.

## Conclusions

WS is a hereditary neurodevelopmental disorder resulting from the haploinsufficiency of genes in the WSCR. WS patients exhibit complex cognitive, behavioral, and neuroanatomical phenotypes. However, sufficient treatments for the neurodevelopmental impairments in WS are still unavailable. Unrevealing the underlying mechanism and promoting curative treatments are of great importance in WS research. Multiple disease models for WS have advanced both mechanistic exploration and therapeutic discovery. Multigene deletion models of WS, CD mice in particular, have enabled systematic investigation of the underlying mechanisms in vivo and facilitated preclinical therapeutic screening. Forebrain organoids capture human-specific neurodevelopmental processes, revealing alterations in neural progenitor dynamics, synaptic maturation, and ribosome biogenesis that are not fully recapitulated in animal models. In addition, single-gene deletion models make it possible to better understand the genotype–phenotype correlations of individual genes in the WSCR. Advances in single-cell and spatial multiomics, optogenetics, and organ-on-a-chip systems will enhance model fidelity and translatability. Ultimately, combining animal- and human-derived systems with precision genetic approaches holds promise for moving beyond symptomatic management toward mechanism-based treatment strategies that can significantly improve outcomes in WS patients.

## Data Availability

This article synthesizes previously published studies and generates no new datasets. All data cited are available in the published articles listed in the references.
